# Large, Rapid Swelling of High-*cis* Polydicyclopentadiene Aerogels Suitable for Solvent-Responsive Actuators

**DOI:** 10.3390/polym12051033

**Published:** 2020-05-02

**Authors:** Despoina Chriti, Grigorios Raptopoulos, Benjamin Brandenburg, Patrina Paraskevopoulou

**Affiliations:** 1 Laboratory of Inorganic Chemistry, Department of Chemistry, National and Kapodistrian University of Athens, Panepistimiopolis Zografou, 15771 Athens, Greece; chritides@chem.uoa.gr (D.C.); grigorisrap@chem.uoa.gr (G.R.); 2 Dräger Safety AG & Co. KGaA, Revalstraße 1, 23560 Lübeck, Germany; Benjamin.Brandenburg@draeger.com

**Keywords:** tungsten, metal-metal bonds, ROMP, dicyclopentadiene, aerogels, solvent-responsive, swelling

## Abstract

High-*cis* polydicyclopentadiene (**PDCPD**) aerogels were synthesized using ring opening metathesis polymerization (ROMP) of dicyclopentadiene (**DCPD**) with a relatively air-stable ditungsten catalytic system, Na[W_2_(*μ*-Cl)_3_Cl_4_(THF)_2_]·(THF)_3_ (**W_2_**; (W^3^W)^6+^, *a′*^2^*e′*^4^), and norbornadiene (**NBD**)as a co-initiator. These aerogels are compared in terms of chemical structure and material properties with literature **PDCPD** aerogels obtained using well-established Ru-based alkylidenes as catalysts. The use of **NBD** as a co-initiator enhances the degree of crosslinking versus the more frequently used phenylacetylene (**PA**), yielding materials with a controlled molecular structure that would persist solvent swelling. Indeed, those **PDCPD** aerogels absorb selected organic solvents (e.g., chloroform, tetrahydrofuran) and swell rapidly, in some cases up to 4 times their original volume within 10 min, thus showing their potential for applications in chemical sensors and solvent-responsive actuators. The advantage of aerogels versus xerogels or dense polymers for these applications is their open porosity, which provides rapid access of the solvent to their interior, thus decreasing the diffusion distance inside the polymer itself, which in turn accelerates the response to the solvents of interest.

## 1. Introduction

Aerogels are usually high-surface-area and extremely low-density materials, and they have become attractive for a wide range of applications, including thermal insulators [1,2,3,4], acoustic insulators [5,6], batteries [7,8], hydrogen storage [9], biomedicine [3,10,11,12,13,14], foods [3,15], sorbents [13,16,17,18], catalysts and catalyst supports [3,17,19,20,21,22]. The last decade has experienced an unprecedented growth of the types of aerogels available, ranging from inorganic [22,23,24,25] to organic (based on biopolymers [3,14,26,27,28] and synthetic polymers) and hybrid inorganic-organic aerogels [29,30,31,32,33,34]. The growth of synthetic polymer aerogels in particular has been extremely rapid and now that class includes aerogels based on a wide variety of phenolic resins [35,36,37], polyamides [38,39,40], polyimides [41,42,43,44,45,46,47], polyurethanes [48,49,50,51,52,53], polyureas [54,55,56,57,58] and polymers derived via ring opening metathesis polymerization (ROMP) [42,49,59,60,61,62,63,64,65].

**Scheme 1:**
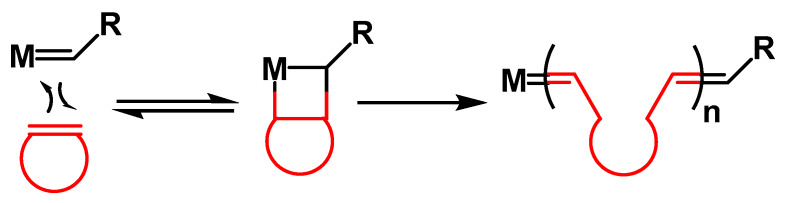
Mechanism of ring-opening metathesis polymerization (ROMP).

**Scheme 2:**
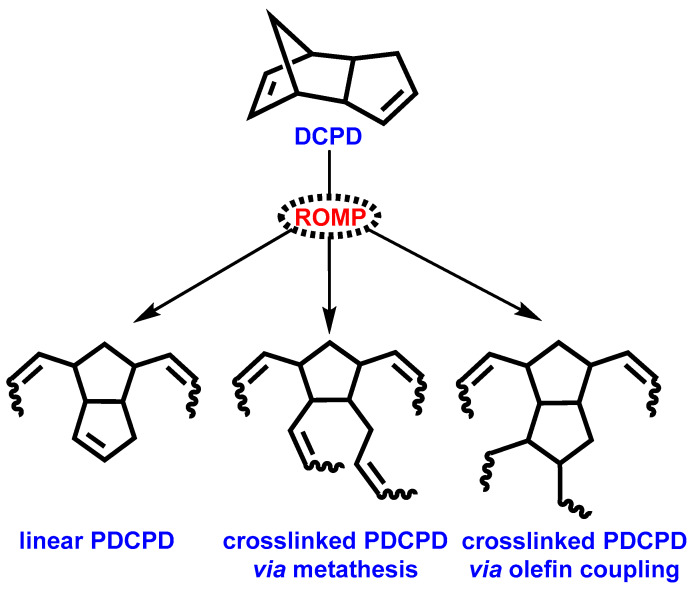
ROMP of **DCPD** including two possible structures from crosslinking.

ROMP (Scheme 1) belongs to a family of metathesis reactions that forms unsaturated, conjugated and non-conjugated polymers. It is considered as one of the most important synthetic tools in polymer and materials science and has been used for the industrial production of several commercial polymers [66,67,68,69,70]. Among them, polydicyclopentadiene (**PDCPD**), a crosslinked polymer prepared from the ROMP of dicyclopentadiene (**DCPD**), is particularly attractive because, among other things, the DCPD monomer is an inexpensive byproduct of the oil industry. As shown in Scheme 2, two mechanisms of crosslinking contribute to the formation of **PDCPD** either via metathesis or via radical addition to the double bonds of the pendant cyclopentene rings [71]. Such crosslinked **PDCPD** is a rigid polymer with excellent mechanical, chemical and physical properties. **PDCPD** aerogels combine the unique properties of aerogels with those of **PDCPD** polymers [59,60,61,62,63,64,65]. Thus, mechanically strong **PDCPD**-based aerogels can find applications as thermal and acoustic insulators [60], as well as low-density coatings [59,64].

A wide variety of catalysts have been reported in the literature for the ROMP of **DCPD**. Most of those catalysts are based on mononuclear complexes of transition metals [59,61,72,73,74], which can be catalytically active per se, or after activation with a co-initiator. Although less widely known, bimetallic clusters with metal-metal bonds have also been employed as ROMP catalysts. For example, both Na[W_2_(*μ*-Cl)_3_Cl_4_(THF)_2_]·(THF)_3_ (**W_2_**; (W^3^W)^6+^, *a′*^2^*e′*^4^) [75,76,77,78,79] and (Ph_4_P)_2_[W_2_(*μ*-Br)_3_Br_6_] (**W_2_-Br**; (W^2.5^W)^7+^, *a′*^2^*e′*^3^) [80] show high catalytic activity towards the metathesis polymerization of alkynes, as well as the polymerization of many cycloolefins. **W_2_**, in particular, yields polymers with a high-*cis* double bond content [75,76,77,78,79]. The addition of phenylacetylene (**PA**) as a co-initiator improves the catalytic activity, while the high-*cis* stereoselectivity is retained [75,78]. Regarding **PDCPD**, the subject matter of this report, **W_2_**-based catalytic systems do provide highly crosslinked and mostly-*cis* polymers [75,78,81]. Owing to their mostly-*cis* structure, **PDCPD** xerogels obtained with the **W_2_/PA** catalytic system have shown an ability for extreme volumetric swelling in various organic solvents (up to more than 100 × the volume of the original xerogel), and selective solvent uptake has been reported from both miscible and immiscible mixtures [75,81].

**Scheme 3:**
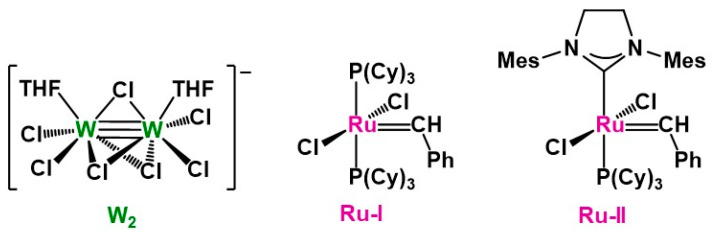
The structures of Na[W_2_(*μ*-Cl)_3_Cl_4_(THF)_2_]·(THF)_3_ (**W_2_**), Grubbs catalyst 1st (**Ru-I**) and 2nd generation (**Ru-II**).

In this study, we describe the synthesis and characterization of **PDCPD** aerogels via ROMP of **DCPD** using **W_2_** as catalyst and norbornadiene (**NBD**) as co-initiator. Similar to **PA**, **NBD** can enhance the activity of **W_2_**, and polymerization with either **PA** or **NBD** as co-initiator proceeds faster. In contrast to **PA**, however, **NBD** has the advantage of yielding crosslinked polymers, while the **W_2_/PA** system yields polymer chains that contain linear polyphenylacetylene segments. Thus, for example, **W_2_/NBD** catalyzes the ROMP of norbornene yielding materials with a controlled molecular structure (star-shaped structure) via the “core-first” synthesis method [79], which is not possible with the **W_2_/PA** system. Another advantage of the higher degree of crosslinking obtained with the **W_2_/NBD** catalytic system is expected to be some rubber-like elasticity that would hold the resulting polymer together after solvent swelling, which in turn would increase reversibility in applications such as sensors and actuators (see next paragraph). In this paper, the configuration and material properties of **PDCPD** aerogels synthesized using the **W_2_/NBD** catalytic system are compared to those of **PDCPD** xerogels and aerogels synthesized not only with the **W_2_/PA** catalytic system [75,81], but also with first and second generation Grubbs’ catalysts (**Ru-I** and **Ru-II**, respectively—Scheme 3), which are known to yield mostly *trans* **PDCPD** [63,75].

At last, we have shown recently that mostly-*cis* **PDCPD** xerogels synthesized with the **W_2_/PA** catalytic system have the ability to absorb large amounts of selected organic solvents (neat or from mixtures) and swell a lot, sometimes up to >100 × the volume of the dry xerogel [75,81]. This property was correlated with the mostly-*cis* structure of the polymer chain, **Ru-I**- and **Ru-II**-derived **PDCPD** aerogels with a significant *trans*-content swell less or do not swell at all [75,81]. In continuation of that study, and because of the growing interest in using crosslinked polymers in the form of gels in chemical sensors and actuators [82,83,84,85,86,87], herein we evaluate highly-crosslinked mostly-*cis* **PDCPD** aerogels synthesized using the catalytic system **W_2_/NBD** for potential use in sensors and solvent-responsive actuators. The advantage of aerogels versus xerogels or dense polymers for these applications is their open porosity, which provides rapid access of the solvent to their interior, which in turn decreases the diffusion distance inside the rigid polymer itself, thus accelerating the response to the solvents of interest.

## 2. Materials and Methods

### 2.1. Materials and Physical Measurements

**DCPD** and **NBD** were purchased from Sigma-Aldrich (Saint louis, MO, USA). **W_2_** was prepared according to literature procedures [88]. **DCPD** and **NBD** were distilled over CaH_2_ under vacuum. Dichloromethane was distilled over P_4_O_10_ and was degassed using three freeze-pump-thaw cycles. Solvents for washings of wet-gels (tetrahydrofuran (THF), pentane and acetone) and for all swelling experiments were used as received. The synthesis of **W_2_** and the preparation of the sol (**W_2_**, **NBD**, **DCPD**, CH_2_Cl_2_) were performed under a pure argon atmosphere, using Schlenk techniques on an inert gas/vacuum manifold or in a drybox (O_2_, H_2_O < 1 ppm).

Attenuated Total Reflection Fourier Transform IR (ATR-FTIR) spectra (525–4000 cm^−1^) were measured on a Fourier-transform instrument (Equinox 55 by Bruker GmbH, Ettlingen, Germany) equipped with a single-reflection diamond ATR accessory (DuraSamplIR II by SensIR Technologies, currently Smiths Detection, Edgewood, MD, USA). Contact between the powder samples and the tip was ensured by applying a suitable level of pressure. The spectra were obtained at an optical resolution of 4 cm^−1^ and are averages of 100 scans. FT-Raman spectra were obtained on a Fourier-transform instrument (RFS 100 by Bruker Optics) employing for excitation ca. 300 mW of the Nd:YAG 1064 nm line in a backscattering geometry. The spectra were obtained at a resolution of 4 cm^−1^ and are averages of ca. 5000–8000 scans. ^13^C cross-polarization magic angle spinning (CPMAS) NMR spectra were obtained with a 600 MHz Varian spectrometer (Varian, Palo Alto, CA, USA) operating at 150.80 MHz for ^13^C. The sample spinning rate used was 5 KHz and the temperature was set at 25 °C.

Thermogravimetric analysis (TGA) was conducted with a Mettler-Toledo TGA (Schwerzenbach, Switzerland), using alumina crucibles. An empty alumina crucible was used as a reference. Samples were heated from ambient temperature to 800 °C in a 50 mL/min flow of N_2_ at a heating rate of 10 °C/min.

N_2_-sorption measurements were made on a Micromeritics Tristar II 3020 surface area and porosity analyzer (Micromeritics, Norcross, GA, USA). Skeletal densities (*ρ*_s_) were determined by He pycnometry, using a Micromeritics AccuPyc II 1340 pycnometer (Micromeritics, Norcross, GA, USA). Bulk densities (*ρ*_b_) of the samples were calculated from their mass and natural dimensions.

Supercritical fluid (SCF) CO_2_ drying was carried out in an autoclave (E3100, Quorum Technologies, East Sussex, UK). Wet-gels were placed in the autoclave at 12 °C and were covered with acetone. Liquid CO_2_ was allowed to flow in the autoclave and at the same time acetone was removed making sure that the samples were always submerged under liquid. The process was repeated 5 times, once every 30 min. Afterwards, the temperature of the autoclave was raised to 45 °C and was maintained for 1 h. Finally, the pressure was gradually released, allowing SCF CO_2_ to escape as a gas, leaving dry-gels (aerogels).

Scanning electron microscopy (SEM) was carried out on dried aerogel samples coated with Au and attached on a conductive double-sided adhesive carbon tape, using a Jeol JSM 5600 SEM instrument (Tokyo, Japan). The system was operating at 20 kV, 0.5 nA and 50 s time of analysis.

### 2.2. Synthesis of **PDCPD** Xerogels and Aerogels Using the Catalytic System **W_2_/NBD**

All formulations are shown in Table S1. In a typical procedure, **NBD** was added to a solution of **W_2_** in dichloromethane, followed by the addition of **DCPD**. The mixture was stirred vigorously at room temperature for 1 min and then was poured into polypropylene molds. All solutions gelled within 15 min. The resulting wet-gels were aged in their molds for 24–28 h at room temperature. Subsequently, wet-gels were removed from their molds and were solvent-exchanged with THF (4×, 8 h per wash cycle, 4× the volume of the gels) and then with (a) pentane (4×, 8 h per wash cycle, 4× the volume of the gels) and they were dried in the oven at 50 °C for 4 h to provide **PDCPD** xerogels or (b) acetone (4×, 8 h per wash cycle, 4× the volume of the gels) and they were dried from SCF CO_2_ to provide **PDCPD** aerogels.

### 2.3. Study of the Swelling Behavior of **PDCPD** Aerogels

Thin disks (0.9–1.0 cm in diameter and 1–2 mm thick) of **PDCPD** aerogels of 0.1 mL initial volume were placed in graduated closed glass tubes, each containing 5 mL of an organic solvent (toluene, dichloromethane, chloroform, chlorobenzene, bromobenzene, THF, 1-bromobutane, ethyl bromide, ethylene dichloride, *m*-xylene, *p*-xylene and mesitylene). They were kept in the solvent for 2 h. The volume increase of the wet-gels in each solvent was measured every 10 min for the first hour and it was determined by the volume decrease of each solvent when the wet-gel was taken out from the graduated glass tube.

## 3. Results and Discussion

### 3.1. Preparation of **PDCPD** Xerogels and Aerogels Using the Catalytic System **W_2_/NBD**

**Scheme 4:**
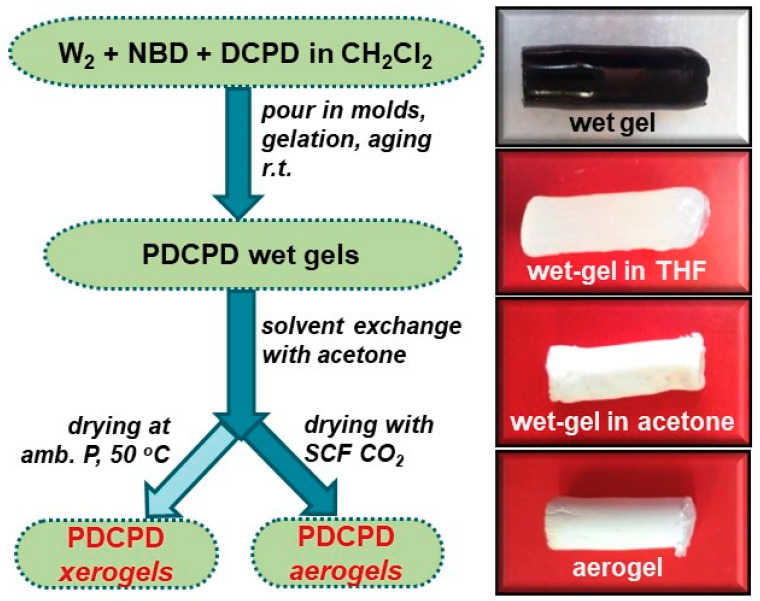
Synthetic route for **PDCPD** sol-gel materials (xerogels and aerogels) from the ROMP of **DCPD** with **W_2_/NBD**. This work is focusing on aerogels. Representative photographs of monoliths (0.9–1.0 cm in diameter, 2.5–3.0 cm in length) at different stages of processing are shown on the right.

The synthesis of **PDCPD** wet-gels was carried out at room temperature under Ar atmosphere (Scheme 4). Wet-gels were either dried in the oven at 50 °C for 4 h or with SCF CO_2_, yielding **PDCPD** xerogels and aerogels, respectively. All formulations are shown in Table S1. The weight percent of **DCPD** was the same for all samples (20% *w/w*). Higher concentrations of monomer provided materials with bulk densities >1 g cm^−3^. Attempts to work with lower concentration sols (e.g., 10 or 5% of **DCPD**) provided wet-gels (within 24 to 48 h) that were not very sturdy and could not be handled easily during post-gelation solvent exchange. The most likely explanation of this behavior is the higher amount of linear **PDCPD**, which is soluble in common organic solvents. Previous works by both our group and others [71,89,90] have shown the formation of linear **PDCPD** in low **DCPD** concentrations. Unreacted monomer and soluble oligomers in the wet-gels were dissolved away during post-gelation solvent exchanges with THF (as shown from the ^1^H NMR spectra of the washes). Different molar ratios of **W_2_/NBD** were tested for a constant concentration of the monomer (20% *w/w*): 1/5, 1/10, 1/20, 1/30 and 1/40. Experiments with a molar ratio of **W_2_/NBD** equal to 1/5 did not gel, while all other ratios provided sturdy wet-gels. By comparison of the material properties of **PDCPD** aerogels (Table 1), the optimal **W_2_/NBD** molar ratio was 1/10. Thereby, all subsequent work with that catalyst was carried out with that molar ratio.

### 3.2. Physicochemical Characterization of **PDCPD** Xerogels and Aerogels

**Figure 1:**
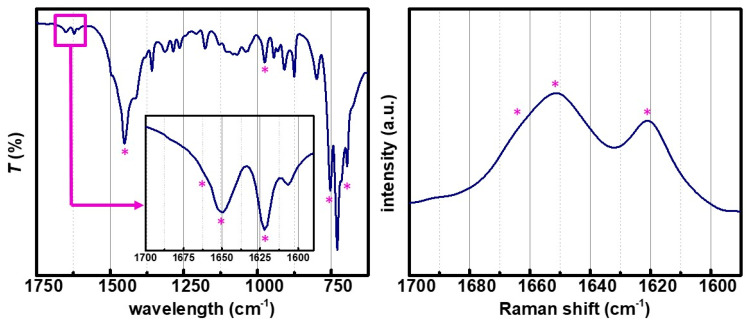
ATR-FTIR (**left**) and FT-Raman (**right**) spectra of **PDCPD** aerogels obtained from the ROMP of **DCPD** with **W_2_/NBD**.

**Figure 2:**
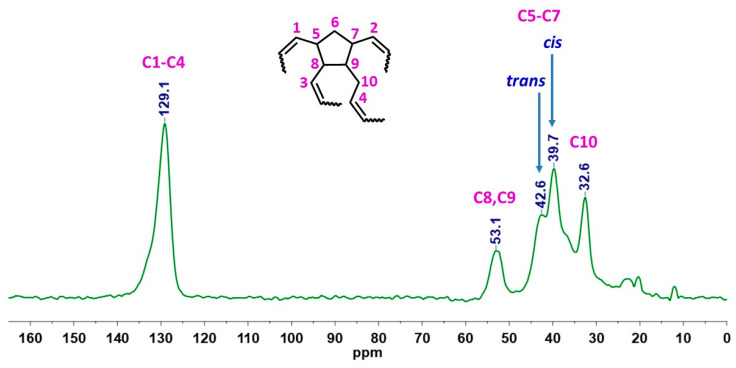
^13^C CPMAS NMR spectrum of **PDCPD** aerogels obtained from the ROMP of **DCPD** with **W_2_/NBD**.

The structure and the configuration of the polymeric chain of **PDCPD** xerogels and aerogels were studied with spectroscopic techniques (ATR-FTIR and FT-Raman—Figure 1 and ^13^C CPMAS NMR—Figure 2) and their thermal stability with thermogravimetric analysis (TGA). As expected, xerogels and aerogels are chemically identical. Therefore, the characterization data for **PDCPD** aerogels are presented below, along with SEM and N_2_ sorption data, which provided information about the porous network of the aerogels. As expected, the chemical characterization showed that the **PDCPD** aerogels of this study were very similar with **PDCPD** xerogels obtained previously with **W_2_/PA** [75], following the characteristic features of reactivity of **W_2_** [75,76,77,78,79], and confirmed the high-*cis* content of the polymer chain. For comparison purposes, figures showing the spectra of **PDCPD** materials obtained with catalytic systems **W_2_/NBD**, **W_2_/PA** (which provides mostly-*trans* **PDCPD)**, and **Ru-I** (which provides mostly-*trans* **PDCPD**) [63,75] are given in the Supporting Information (Figure S1 and Figure S2).

More specifically, ATR-FTIR spectra (Figure 1 and Figure S1, top) showed the stretching vibration of *trans* and *cis* C=C bonds at 1660 cm^−1^ and at 1650 cm^−1^, respectively, and deformation vibrations of C–H bonds on *trans* and *cis* double bonds at 976 and 750 cm^−1^, respectively. A shoulder at 710 cm^−1^ proved the existence of unreacted pendant cyclopentene groups, pointing out the presence of linear **PDCPD** segments in the polymer backbone. The relative intensity of the bands related to *cis/trans* bonds, in comparison to the spectra of **PDCPD** obtained with catalytic systems **W_2_/PA** and **Ru-I** (Figure S1, top), suggested that **W_2_/NBD** and **W_2_/PA** provided **PDCPD** with the same configuration, i.e., mostly-*cis* [75]. In addition, Raman spectra of both materials were almost identical (Figure 1 and Figure S1, bottom), showing two characteristic bands at 1650 and 1622 cm^−1^, which were attributed to the acyclic *cis* double bonds of the polymer network and to the cyclic *cis* cyclopentene double bonds, respectively, and a small shoulder at 1664 cm^−1^, corresponding to the *v*(C=C) of the *trans* double bonds of the polymeric chain [75,91,92].

In agreement with ATR-FTIR and FT-Raman spectroscopy, ^13^C CPMAS NMR spectroscopy (Figure 2 and Figure S2) also confirmed the high-*cis* configuration of **PDCPD** aerogels. The peak at 40 ppm was assigned to *cis* double bonds of the polymeric chain and prevailed over the peak at 44 ppm, which was assigned to *trans* double bonds of the polymeric chain [63,75]. The exact determination of the *cis*/*trans* ratio was not possible due to overlapping of the two peaks. However, the stereoselectivity of each catalytic system is rather straightforward (Figure 2).

The thermal stability of the **PDCPD** aerogels was investigated using thermogravimetric analysis (TGA) under nitrogen (Figure S3, left). TGA curves for materials obtained with the three catalytic systems were very similar and showed that the thermal decomposition can be divided into two steps. For the **PDCPD** aerogels of this study, a very small weight loss (2%) was observed during the first step, from 25 to 420 °C, corresponding to the evaporation and decomposition of unreacted monomers and oligomers. The second and main degradation step happened after 460 °C and resulted in a residue of 18%. As can be seen in differential thermogravimetry (Figure S3, right), a shoulder appeared at 470 °C, indicating a bimodal and more complex thermal decomposition mechanism than in the case of **PDCPD** obtained with **Ru-I**.

**Figure 3:**
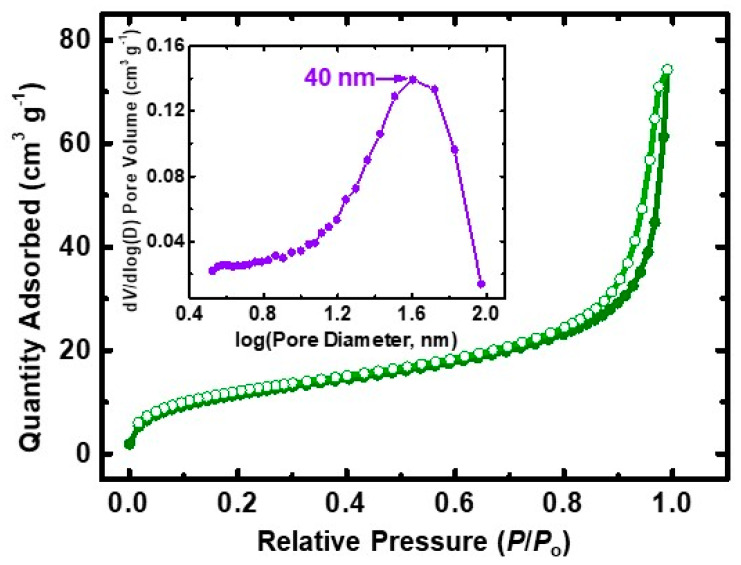
N_2_-sorption isotherm for **PDCPD** aerogels obtained from the ROMP of **DCPD** with **W_2_/NBD** (Table 1, entry 1). Inset: pore size distribution using the BJH method.

**PDCPD** xerogels synthesized with **W_2_/NBD** had no porosity or little porosity (for example, for a molar ratio of **W_2_/NBD** equal to 1/10, the bulk density was around 1 g cm^−3^ and the skeletal density was 1.1 g cm^−3^, therefore the porosity was only 9% *v/v*). Therefore, and despite their more straightforward preparation, **PDCPD** xerogels were not considered further for this work. Selected material properties for **PDCPD** aerogels are summarized in Table 1. Lower bulk density, higher porosity, higher BET (Brunauer–Emmett–Teller) surface area and smaller particle sizes were obtained for **PDCPD** aerogels synthesized using the lowest **W_2_/NBD** molar ratio (1/10). The skeletal density was also lower, compared to the higher **W_2_/NBD** molar ratios, as a result of the incorporation of more or longer polynorbornadiene (**PNBD**) segments in the polymer chain. The shape of the N_2_-sorption isotherm (i.e., no saturation, narrow hysteresis loop; Figure 3) and the fact that *V*_Total_ >> *V*_1.7–300nm_ (Table 1) indicate that our materials are macroporous, in agreement with the literature [63]. Average pore diameters were calculated using the 4 *V*/*σ* method. *V* was set either as the maximum volume of N_2_ adsorbed along the isotherm or as the volume (*V*_Total_) calculated from the bulk and the skeletal density of the corresponding materials (Table 1). Average pore diameter using *V*_Total_ were higher and they increased with decreasing bulk density. From the BJH (Barrett-Joyner-Halenda) method, the peak maximum was at 40 nm.

Table 1 also includes selected material properties of **PDCPD** aerogels from the literature [63], synthesized with **Ru-I** and **Ru-II**. All comparisons are made for aerogels from sols of the same concentration (20% *w/w*). **PDCPD** aerogels of this study (**W_2_/NBD** 1/10) have lower bulk density and higher porosity, but significantly lower BET surface area compared to **PDCPD** aerogels from **Ru-I**. On the other hand, they have similar BET surface area (42 vs. 38 m^2^/g) and particle size (63 vs. 75 nm), and lower average pore diameter (11 vs. 32 nm) compared to **PDCPD** aerogels from **Ru-II**. It seems that high-*cis* **PDCPD** aerogels (from **W_2_/NBD**) have similar properties to high-*trans* **PDCPD** aerogels (from **Ru-II**), and they have three advantages: (a) they are sturdy and well-shaped (Scheme 4), while **Ru-II**-derived aerogels were deformed, (b) the **W_2_/NBD** catalytic system is more cost-efficient compared to **Ru-II**, and (c) high-*cis* **PDCPD** aerogels can be used for applications related to environmental remediation (see next section).

**Figure 4:**
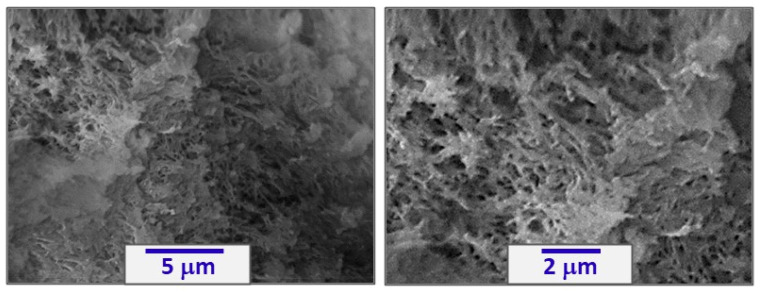
SEM images of **PDCPD** aerogels obtained from the ROMP of **DCPD** with **W_2_/NBD**.

The morphology of **PDCPD** aerogels prepared with **W_2_/NBD** was investigated with SEM, which revealed a beaded fibrous microstructure (Figure 4). That morphology is different from that of **PDCPD** aerogels prepared with the same sol concentration (20% *w/w*) using **Ru-I** or **Ru-II** catalysts [63]. Those materials were macroporous, and although they were also fibrous, no beads could be seen along the fibers even at higher magnifications. It is suggested that this difference in morphology is related to the different *cis*/*trans* configuration of the polymers, which is translated to different mechanisms of phase separation along gelation. Differences in morphologies that could be attributed to the configuration of the polymer chains have also been observed in norbornene/norbornadiene copolymers. Furthermore, high-*cis* copolymers, obtained with **W_2_/PA** [79], had different morphologies than copolymers with a 50/50 *cis*/*trans* double bond ratio, obtained with Ru-based catalysts [93,94,95].

### 3.3. Swelling Studies

We have shown recently that mostly-*cis* **PDCPD** xerogels synthesized with the **W_2_/PA** catalytic system have the ability to absorb large amounts of organic solvents and swell [75,81]. This property is related with the mostly-*cis* structure of the polymer chain, as **PDCPD** xerogels and aerogels that have a significant *trans*-content swell less or do not swell at all [75,81]. **PDCPD** aerogels of this study could also swell in selected organic solvents (e.g., toluene, dichloromethane, THF, chloroform) by absorbing a significant amount of solvent. Compared to **PDCPD** materials obtained with the **W_2_/PA** catalytic system, the **PDCPD** aerogels of this work were sturdier. In the first case, the small chains of polyphenylacetylene were soluble and had to be removed, while in the present case, the small amount of **PNBD** that is formed is insoluble and cannot and does not need to be washed out. The fact that **PDCPD** aerogels derived with the **W_2_/NBD** catalytic system swell significantly, although **PNBD** is a crosslinked polymer, is important and may be attributed to the fact that those materials are not random copolymers, but **PNBD** chains are rather confined at the ends of the **PDCPD** chains. Furthermore, it should be pointed out that said swelling takes place fast, which is attributed to the synergistic effect of the open porosity of the aerogels, which provides rapid access of the solvent to their interior, and therefore the diffusion distance inside the rigid polymer is shortened and the response to the solvents of interest is accelerated.

We evaluated those aerogels versus their potential to be used as sensors. In the literature, there is a growing interest for the use of crosslinked polymers in the form of gels in chemical sensors [82,83,84,85,86,87]. For this kind of application, the swelling behavior of those gels needs to be examined [86,87]. Swelling is essentially a chain rearrangement, resulting from interactions between the polymer and its environment [87], which, in this case, is the solvent. In order for a gel to be used as a sensor, it has to show a fast volume change when exposed to external stimuli. For this fast response to be achieved, the gel dimensions need to be small.

**Figure 5:**
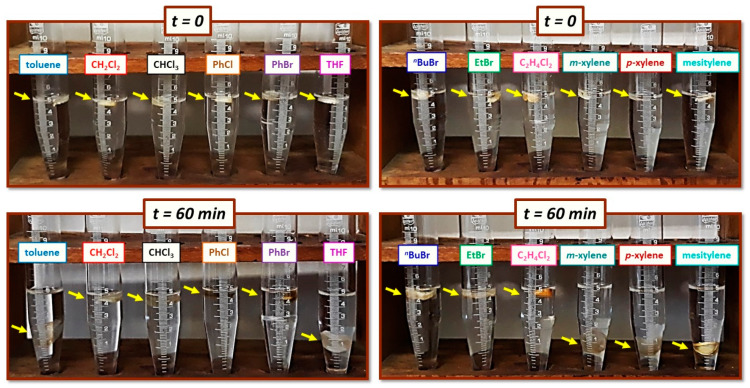
Representative optical photographs at two time intervals (**top**: *t* = 0 min; **bottom**: *t* = 60 min) of graduated tubes containing **PDCPD** aerogel thin disks and various organic solvents, as indicated. Yellow arrows point at the wet-gels.

**Figure 6:**
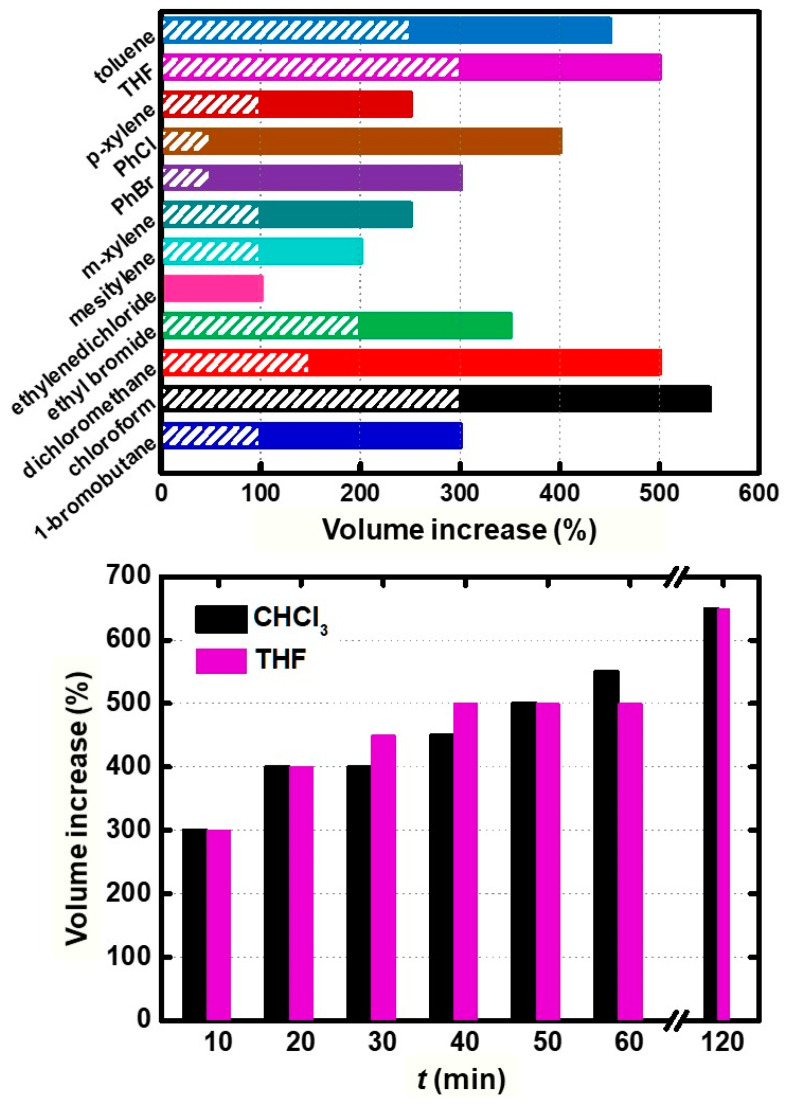
**Top**: Volume increase of thin **PDCPD** aerogel disks in various organic solvents at 10 min (shaded length of each column) and at 60 min (full length of each column). **Bottom**: Comparison of the volume increase of thin **PDCPD** aerogel disks in THF and chloroform within 120 min.

For that reason, **PDCPD** aerogels in the form of thin disks were added in graduated glass tubes containing 5 mL of various neat organic solvents (toluene, dichloromethane, chloroform, chlorobenzene, bromobenzene, THF, 1-bromobutane, ethyl bromide, ethylene dichloride, *m*-xylene, *p*-xylene and mesitylene—Figure 5). The initial volume of the disks was 0.1 mL in all cases. Gels were kept in the solvent for 2 h in total and for the first hour the volume increase (Δ*V*) of gels in each solvent was measured every 10 min. Δ*V* was determined by the volume decrease of each solvent when the gel was taken out of the graduated tube. Results are summarized in Table 2 and graphically represented in Figure 6. All plots (volume increase vs. time) are provided in the Supporting Information (Figures S4–S15). The higher volume increase was observed in chloroform, dichloromethane, THF and toluene, with the fastest response in chloroform and THF (300% volume increase within the first 10 min). In most of the solvents, the volume increase after 2 h did not change too much (Table 2; Figures S4–S15).

## 4. Conclusions

High-*cis* poly(dicyclopentadiene) (**PDCPD**) aerogels were synthesized via ROMP of dicyclopentadiene catalyzed using the ditungsten catalytic system, Na[W_2_(*μ*-Cl)_3_Cl_4_(THF)_2_]·(THF)_3_ (**W_2_**; (W^3^W)^6+^, *a′*^2^*e′*^4^), and norbornadiene (**NBD**) as a co-initiator. High-*cis* **PDCPD** aerogels have several similarities with high-*trans* **PDCPD** aerogels obtained with Ru-based catalysts; for example, they are both macroporous materials with low BET surface areas (42 vs. 38 m^2^/g) and fibrous morphologies. However, the aspect ratios of the two kinds of fibers were different and fibers from the high-*cis* polymer consisted of strings of beads, while fibers of the high-*trans* polymer were smooth, in agreement with our previous observations for high-*cis* and high-*trans* norbornene/norbornadiene copolymers. Most interestingly though, high-*cis* **PDCPD** aerogels absorb selected organic solvents and swell rapidly, in some cases up to 4 times their original volume within 10 min, thus comprising viable candidates for applications in chemical sensors and solvent-responsive actuators. The advantage of aerogels versus xerogels or dense polymers for these applications is their open porosity, which provides rapid access of the solvent to their interior, which decreases the diffusion distance inside the polymer itself and accelerates the response to the solvents of interest.

## Figures and Tables

**Table 1:** Selected material properties of **PDCPD** aerogels obtained from the ROMP of **DCPD** with three different catalytic systems.

Catalytic System (Molar Ratio)	Linear Shrinkage ^1^ (%)	Bulk Density *ρ_b_* (g cm^−3^)	Skeletal Density *ρ_s_* (g cm^−3^)	Porosity ^2^ *Π* (% *v/v*)	BET Surf. Area *σ* (m^2^g^−1^)	*V*_Total_^3^ (*V*_1.7–300nm_) ^4^ (cm^3^ g^−1^)	Av. Pore Diameter ^5^ (nm)	Particle Radius ^6^ (nm)
**W_2_/NBD** **1/10**	15	0.135 ± 0.009	1.128 ± 0.005	88	42	7.0 (0.1)	11 (667)	63
**W_2_/NBD** **1/20**	26	0.52 ± 0.08	1.348 ± 0.006	61	<10	1.6 (0.009)	6.6 (914)	-
**Ru-I** [63]	12	0.28 ± 0.07	1.136 ± 0.003	75	186	2.7 (NA)	21 (57)	14.2
**Ru-II** [63]	NA ^7^	NA	1.055 ± 0.004	-	38	NA (NA)	32 (NA)	75

^1^ Linear shrinkage after SCF drying calculated according to formula (*y*_1_ + *y*_2_)/2, where *y*_1_ = 100 − [(*h*_fin_/*h*_st_) × 100] and *y*_2_ = 100 − [(*d*_fin_/*d*_st_) × 100]; *h*: height, *d*: diameter. ^2^ Porosity calculated according to formula 100 × (*ρ*_s_ − *ρ*_b_)/*ρ*_s_, where *ρ*_s_: skeletal density and *ρ*_b_: bulk density. ^3^ Volume calculated according to formula 1/*ρ*_b_ − 1/*ρ*_s_. ^4^ Cumulative volume of pores between 1.7 and 300 nm from N_2_-sorption data and the BJH desorption method. ^5^ Calculated using the 4*V*/*σ* method; *V* was set equal to the maximum volume of N_2_ adsorbed along the isotherm as *P*/*P*_o_ → 1.0. For the number in parenthesis, *V* was set equal to *V*_Total_ from the previous column. ^6^ Particle size calculated according to formula *r* = 3/(*ρ*_s_ × *σ*). ^7^ Deformed cylinder; not measured.

**Table 2:** Volume increase of **PDCPD** aerogel thin disks (initial volume = 0.1 mL) in various organic solvents.

Solvent												
Time (min)	Toluene	CH_2_Cl_2_	CHCl_3_	PhCl	PhBr	THF	1-Bromo Butane	Ethyl Bromide	Ethylene Dichloride	*Meta*- Xylene	*Para*- Xylene	Mesity- lene
	Volume Increase (Δ*V*, mL)											
**0**	0.00	0.00	0.00	0.00	0.00	0.00	0.00	0.00	0.00	0.00	0.00	0.00
**10**	0.25	0.15	0.30	0.05	0.05	0.30	0.10	0.20	0.00	0.10	0.10	0.10
**20**	0.30	0.35	0.40	0.20	0.20	0.40	0.20	0.20	0.00	0.10	0.15	0.10
**30**	0.30	0.40	0.40	0.30	0.20	0.45	0.20	0.25	0.10	0.10	0.25	0.15
**40**	0.30	0.40	0.45	0.30	0.25	0.50	0.20	0.30	0.10	0.20	0.25	0.15
**50**	0.40	0.40	0.50	0.30	0.30	0.50	0.20	0.30	0.10	0.20	0.25	0.20
**60**	0.45	0.50	0.55	0.40	0.30	0.50	0.30	0.35	0.10	0.25	0.25	0.20
**120**	0.45	0.60	0.65	0.40	0.40	0.65	0.30	0.40	0.10	0.35	0.40	0.30

## Supplementary Materials

The following are available online at https://www.mdpi.com/2073-4360/12/5/1033/s1; Table S1: Formulations used for the synthesis of **PDCPD** xerogels and aerogels using the catalytic system **W_2_/NBD**; Figure S1: Top row: ATR-FTIR spectra (left: 1700–1590 cm^−1^; right: 1500–675 cm^−1^) of **PDCPD** aerogels and xerogels obtained from ROMP of **DCPD** with three catalytic systems, as indicated. Bottom row: FT-Raman spectra (left: 3200–1200 cm^−1^; right: 1710–1590 cm^−1^) of **PDCPD** aerogels and xerogels obtained from the ROMP of **DCPD** with three catalytic systems, as indicated; Figure S2: ^13^C CPMAS NMR spectra of **PDCPD** aerogels obtained from ROMP of **DCPD** with three catalytic systems, as indicated; Figure S3: Weight loss with temperature (left) and derivative weight loss with temperature (right) of **PDCPD** aerogels and xerogels obtained from the ROMP of **DCPD** with three catalytic systems, as indicated; Figure S4: Swelling of a **PDCPD** aerogel thin disk in toluene versus time; Figure S5: Swelling of a **PDCPD** aerogel thin disk in dichloromethane versus time; Figure S6: Swelling of a **PDCPD** aerogel thin disk in chloroform versus time; Figure S7: Swelling of a **PDCPD** aerogel thin disk in chlorobenzene versus time; Figure S8: Swelling of a **PDCPD** aerogel thin disk in bromobenzene versus time; Figure S9: Swelling of a **PDCPD** aerogel thin disk in THF versus time; Figure S10: Swelling of a **PDCPD** aerogel thin disk in 1-bromobutane versus time; Figure S11: Swelling of a **PDCPD** aerogel thin disk in ethyl bromide versus time; Figure S12: Swelling of a **PDCPD** aerogel thin disk in ethylene dichloride versus time; Figure S13: Swelling of a **PDCPD** aerogel thin disk in *m*-xylene versus time; Figure S14: Swelling of a **PDCPD** aerogel thin disk in *p*-xylene versus time; Figure S15: Swelling of a **PDCPD** aerogel thin disk in mesitylene versus time.
