# Supplementary material for: Large, Rapid Swelling of High-cis Polydicyclopentadiene Aerogels Suitable for Solvent-Responsive Actuators
Source: Polymers (Basel). 2020 May 2;12(5):1033. doi: 10.3390/polym12051033 (PMC7284835; doi:10.3390/polym12051033)
Supplement: Supplementary file 1 [file polymers-12-01033-s001.pdf]

Large, rapid swelling of high-*cis* polydicyclopentadiene aerogels suitable for solvent-responsive actuators

Despoina Chriti,[1] Grigorios Raptopoulos,[1] Benjamin Brandenburg,[2] Patrina Paraskevopoulou[1,*]

[1] *Laboratory of Inorganic Chemistry, Department of Chemistry, National and Kapodistrian University of Athens, Panepistimiopolis Zografou, Athens 15771, Greece.*

[2] *Dräger Safety AG & Co. KGaA, Revalstraße 1, 23560 Lübeck, Germany.*

* Correspondance : paraskevopoulou@chem.uoa.gr (P.P.); Tel.: +30-210-727-4381; Fax: +30-210-727-4782

Table of Contents

	Page
**Table of Contents**	S-2
**Table S1.** Formulations used for the synthesis of **PDCPD** xerogels and aerogels using the catalytic system $\mathbf{W_2/NBD}$.	S-3
**Figure S1.** Top row: ATR-FTIR spectra (left: 1700-1590 cm^{-1}; right: 1500-675 cm^{-1}) of **PDCPD** aerogels and xerogels obtained from ROMP of **DCPD** with three catalytic systems, as indicated. Bottom row: FT-Raman spectra (left: 3200-1200 cm^{-1}; right: 1710-1590 cm^{-1}) of **PDCPD** aerogels and xerogels obtained from the ROMP of DCPD with three catalytic systems, as indicated.	S-3
**Figure S2.** ^{13}C CPMAS NMR spectra of **PDCPD** aerogels obtained from ROMP of **DCPD** with three catalytic systems, as indicated.	S-4
**Figure S3.** Weight loss with temperature (left) and derivative weight loss with temperature (right) of **PDCPD** aerogels and xerogels obtained from the ROMP of **DCPD** with three catalytic systems, as indicated.	S-4
**Figure S4.** Swelling of a **PDCPD** aerogel thin disk in toluene *versus* time.	S-5
**Figure S5.** Swelling of a **PDCPD** aerogel thin disk in dichloromethane *versus* time.	S-5
**Figure S6.** Swelling of a **PDCPD** aerogel thin disk in chloroform *versus* time.	S-6
**Figure S7.** Swelling of a **PDCPD** aerogel thin disk in chlorobenzene *versus* time.	S-6
**Figure S8.** Swelling of a **PDCPD** aerogel thin disk in bromobenzene *versus* time.	S-7
**Figure S9.** Swelling of a **PDCPD** aerogel thin disk in THF *versus* time.	S-7
**Figure S10.** Swelling of a **PDCPD** aerogel thin disk in 1-bromobutane *versus* time.	S-8
**Figure S11.** Swelling of a **PDCPD** aerogel thin disk in ethyl bromide *versus* time.	S-8
**Figure S12.** Swelling of a **PDCPD** aerogel thin disk in ethylene dichloride *versus* time.	S-9
**Figure S13.** Swelling of a **PDCPD** aerogel thin disk in *m*-xylene *versus* time.	S-9
**Figure S14.** Swelling of a **PDCPD** aerogel thin disk in *p*-xylene *versus* time.	S-10
**Figure S15.** Swelling of a **PDCPD** aerogel thin disk in mesitylene *versus* time.	S-10

Table S2. Formulations used for the synthesis of **PDCPD** xerogels and aerogels using the catalytic system **W_2/NBD**.[a]

W_2/NBD/DCPD (molar ratio)	Catalyst (mg, mmol)	NBD (μL, mmol)	DCPD[b] (mL, mmol)	Gelation time (min)
1/5/350		10.2, 0.1		-[c]
1/10/350		20.4, 0.2		15
1/20/350	21.50, 0.02	40.8, 0.4	1.00, 7.0	10
1/30/350		61.2, 0.6		5
1/40/350		81.6, 0.8		3

[a] Solvent: methylene dichloride (12 mL). [b] [DCPD] = 20% w/w. [c] No gelation was observed within 72 h.

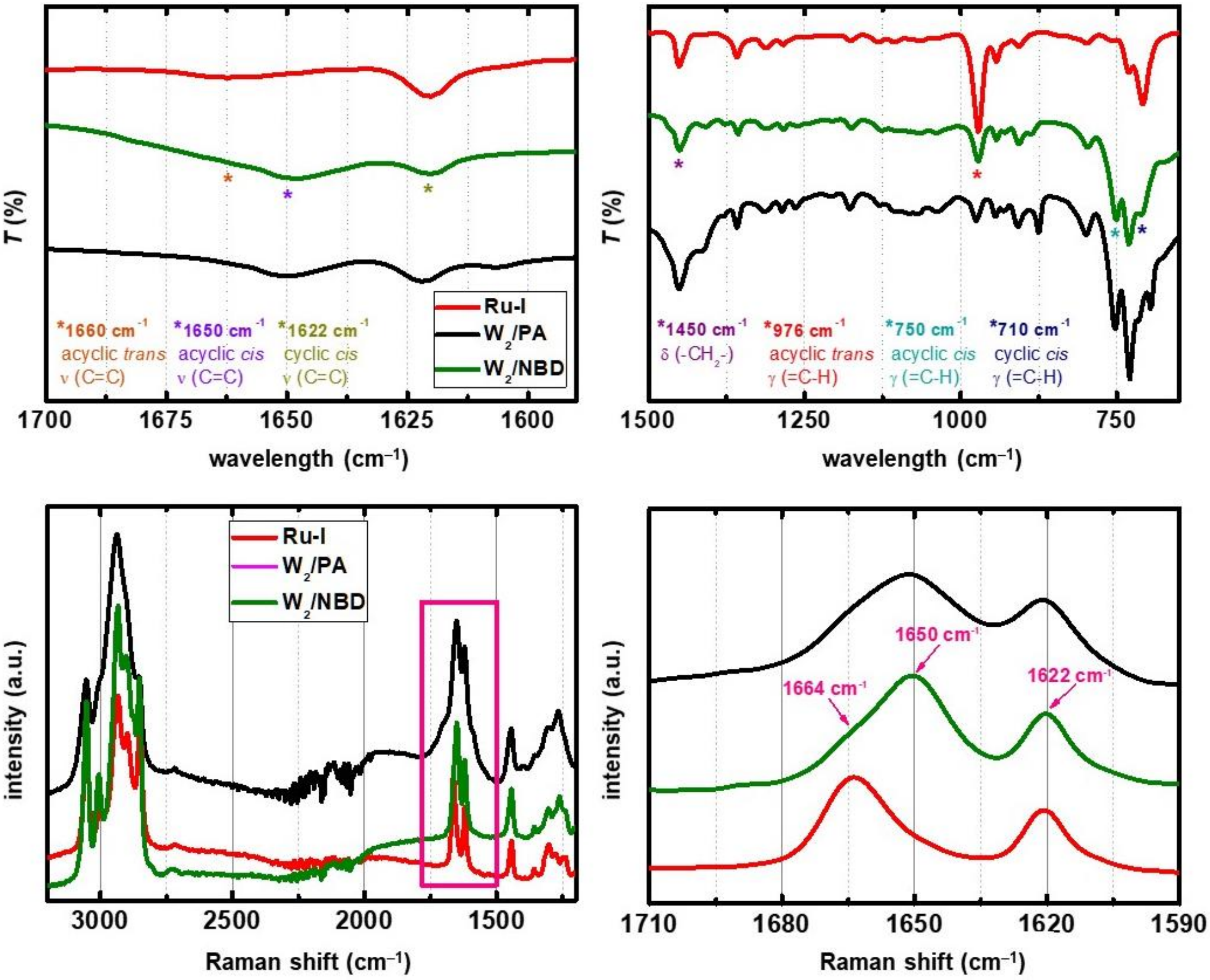

Figure S1. Top row: ATR-FTIR spectra (left: 1700-1590 cm^{-1}; right: 1500-675 cm^{-1}) of **PDCPD** aerogels and xerogels obtained from ROMP of **DCPD** with three catalytic systems, as indicated. Bottom row: FT-Raman spectra (left: 3200-1200 cm^{-1}; right: 1710-1590 cm^{-1}) of **PDCPD** aerogels and xerogels obtained from the ROMP of **DCPD** with three catalytic systems, as indicated.

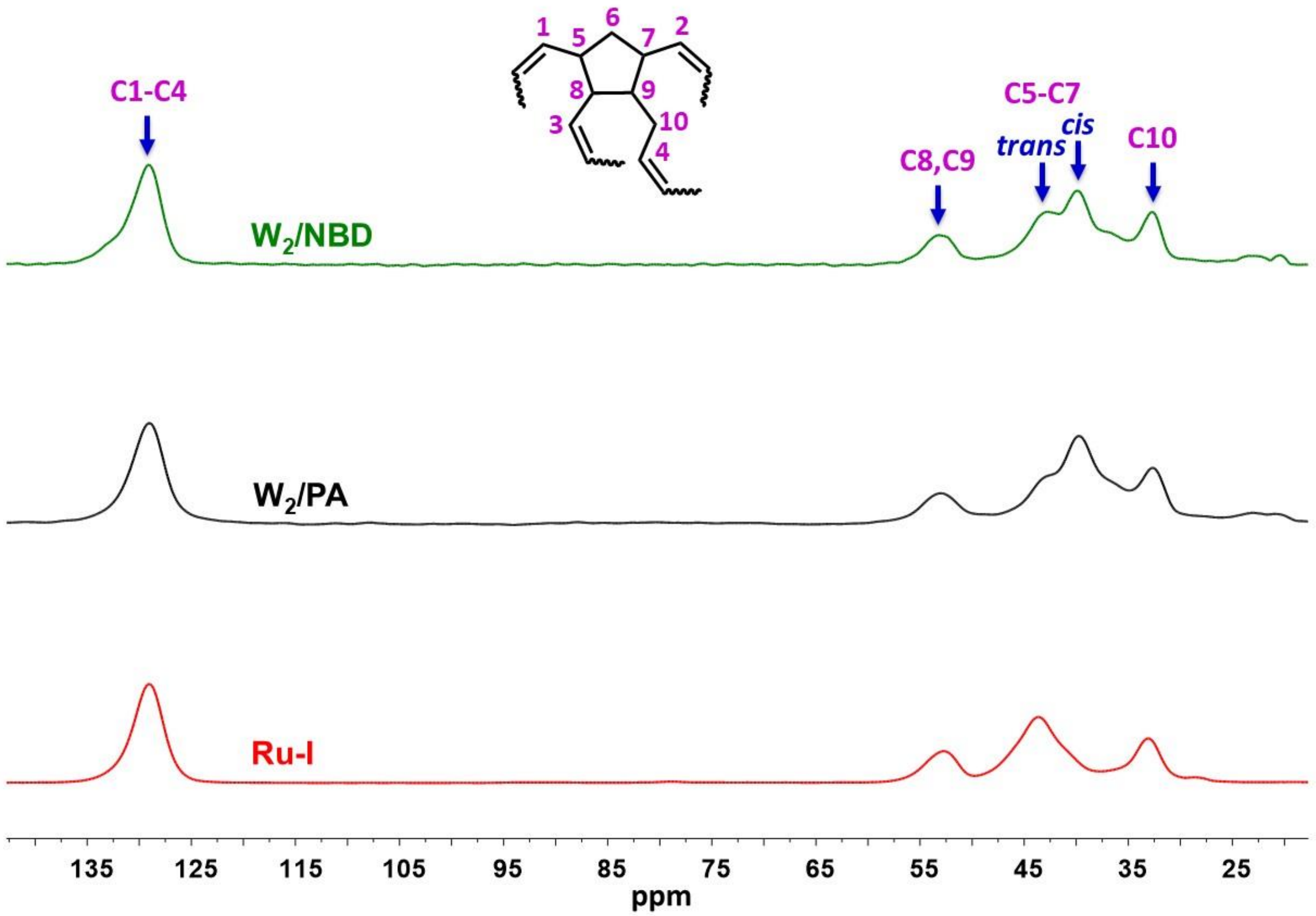

Figure S2. ^{13}C CPMAS NMR spectra of **PDCPD** aerogels obtained from ROMP of **DCPD** with three catalytic systems, as indicated.

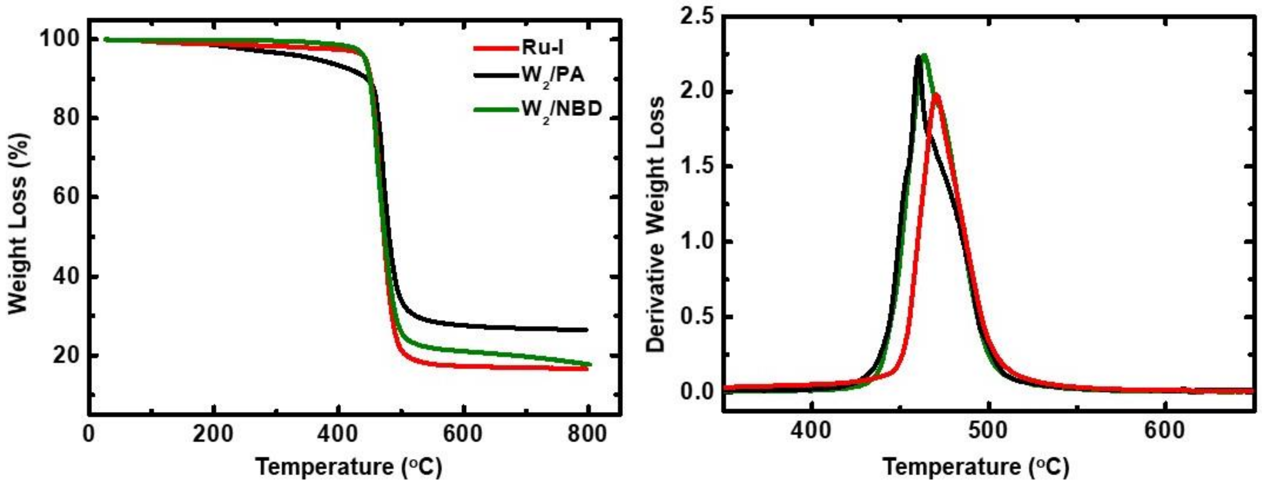

Figure S3. Weight loss with temperature (left) and derivative weight loss with temperature (right) of **PDCPD** aerogels and xerogels obtained from the ROMP of **DCPD** with three catalytic systems, as indicated.

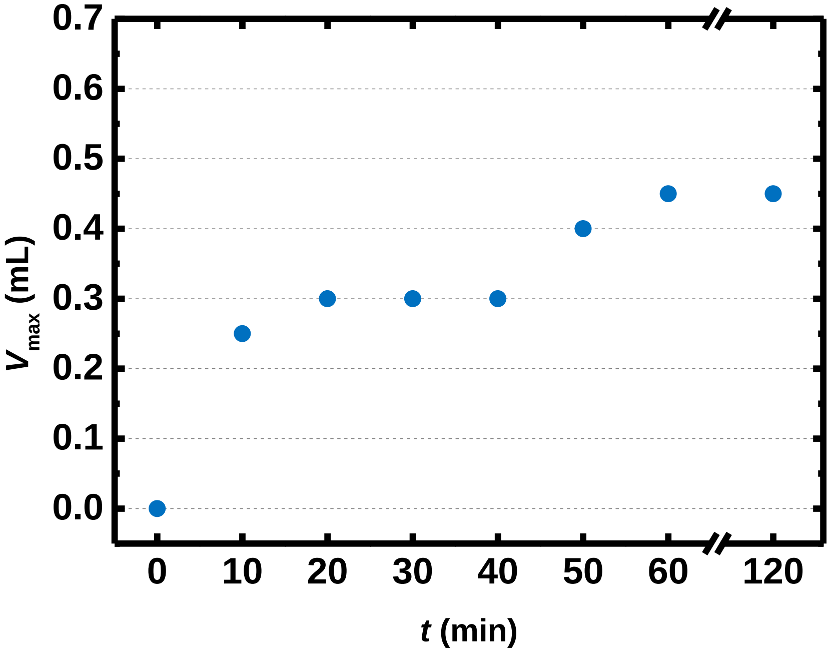

Figure S4. Swelling of a **PDCPD** aerogel thin disk in toluene *versus* time.

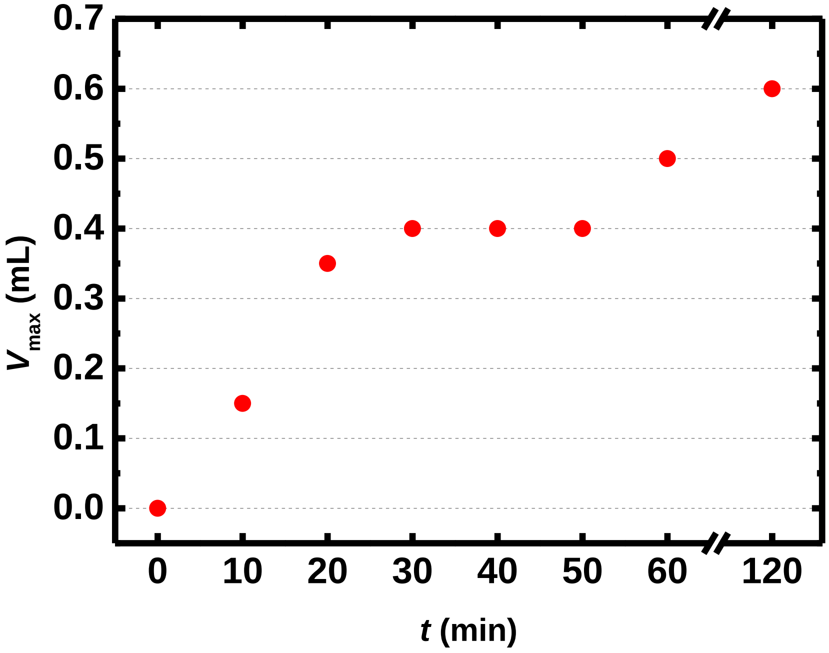

Figure S5. Swelling of a **PDCPD** aerogel thin disk in dichloromethane *versus* time.

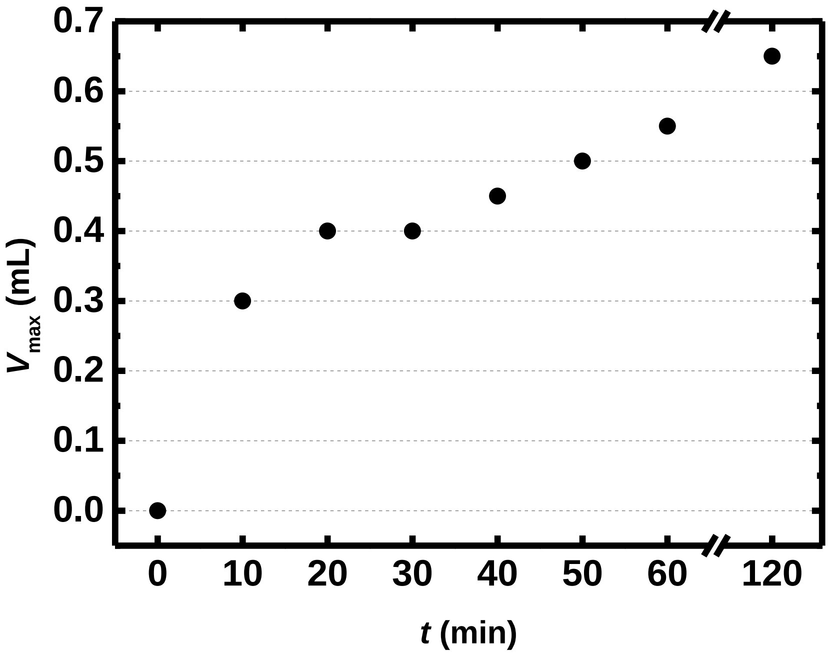

Figure S6. Swelling of a **PDCPD** aerogel thin disk in chloroform *versus* time.

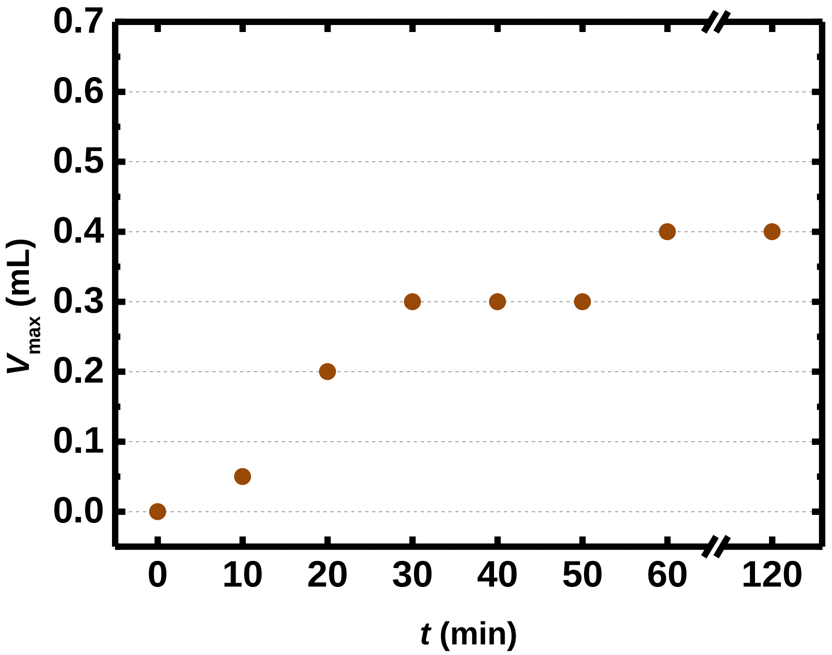

Figure S7. Swelling of a **PDCPD** aerogel thin disk in chlorobenzene *versus* time.

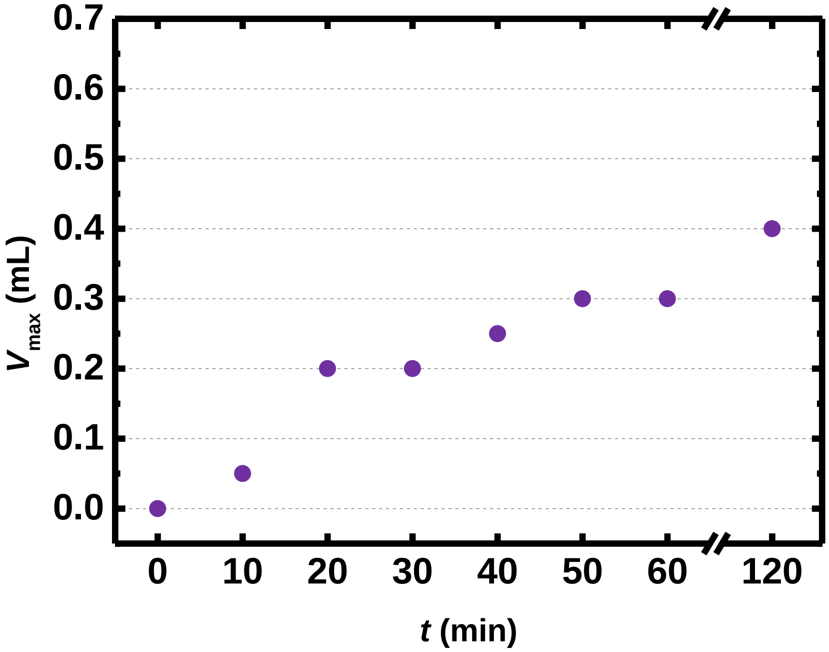

Figure S8. Swelling of a **PDCPD** aerogel thin disk in bromobenzene *versus* time.

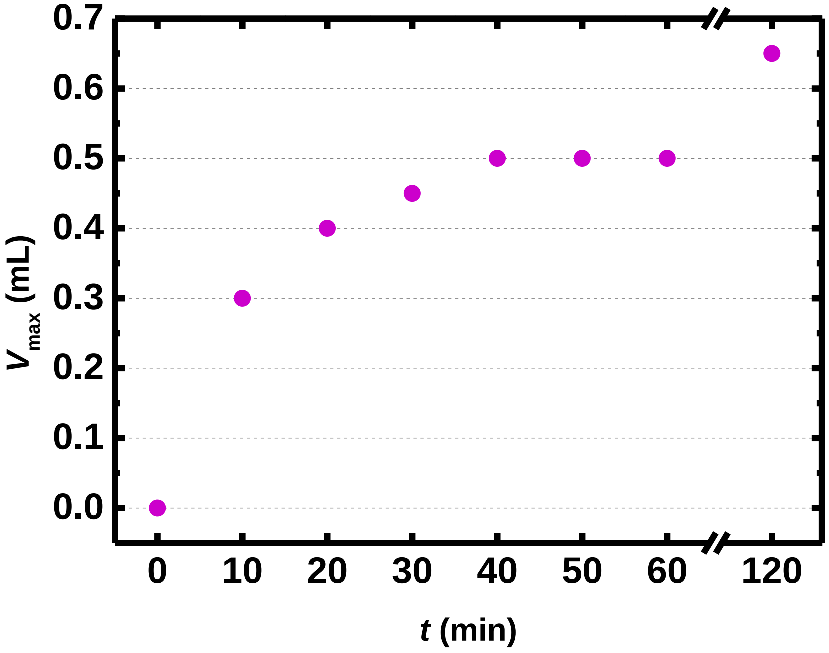

Figure S9. Swelling of a **PDCPD** aerogel thin disk in THF *versus* time.

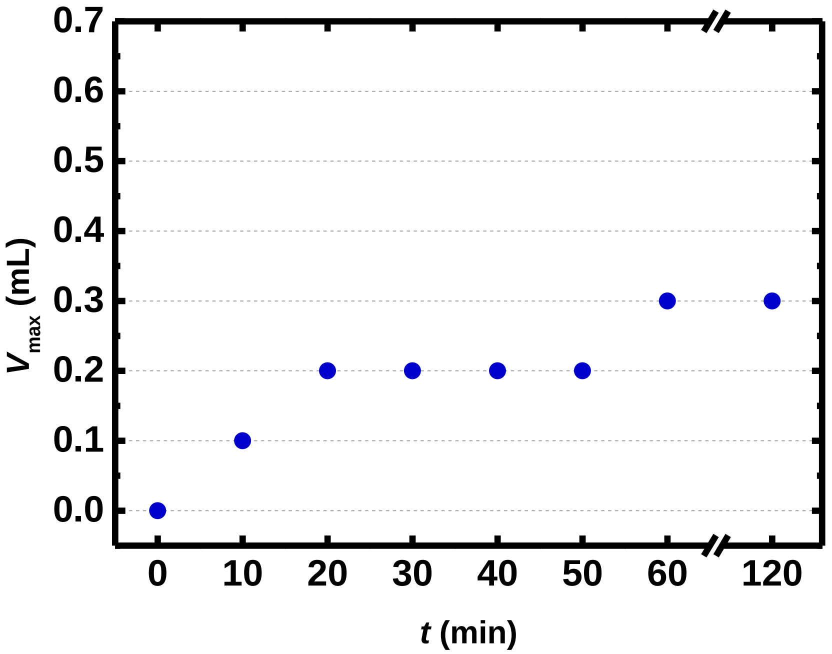

Figure S10. Swelling of a **PDCPD** aerogel thin disk in 1-bromobutane *versus* time.

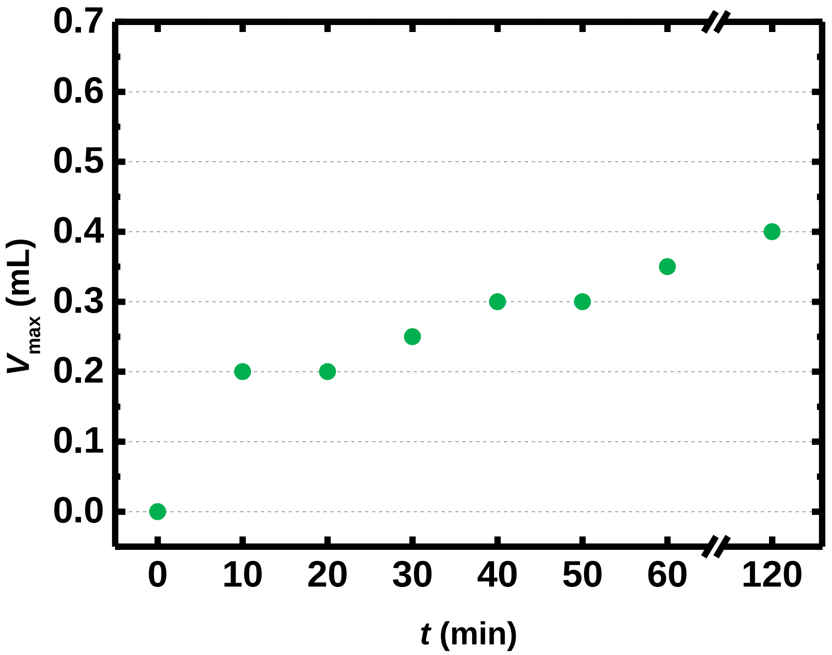

Figure S11. Swelling of a **PDCPD** aerogel thin disk in ethyl bromide *versus* time.

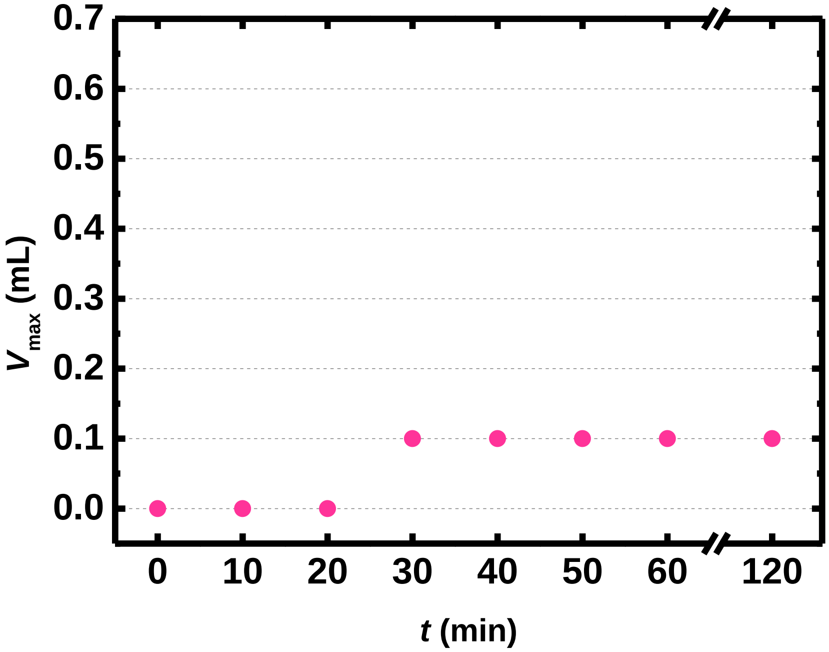

Figure S12. Swelling of a **PDCPD** aerogel thin disk in ethylene dichloride *versus* time.

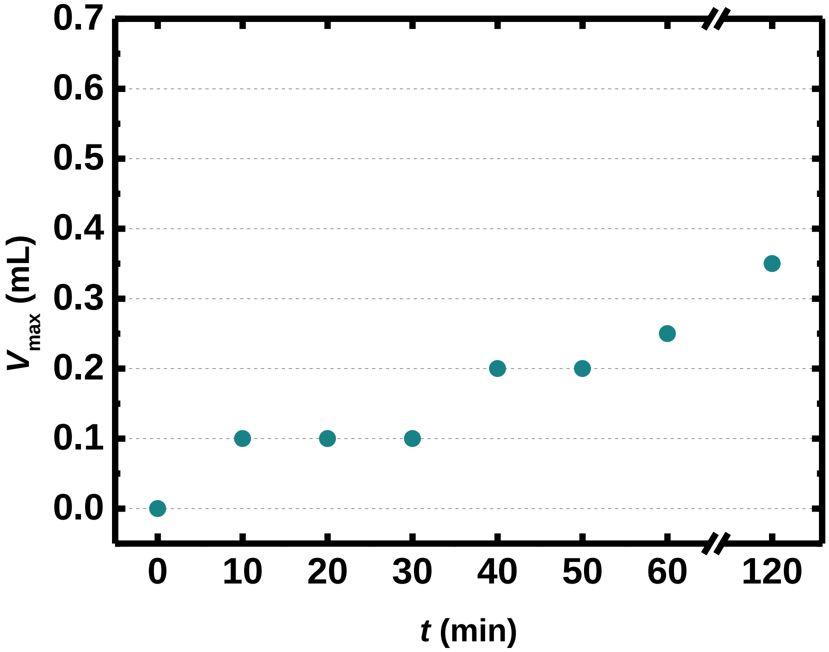

Figure S13. Swelling of a **PDCPD** aerogel thin disk in *m*-xylene *versus* time.

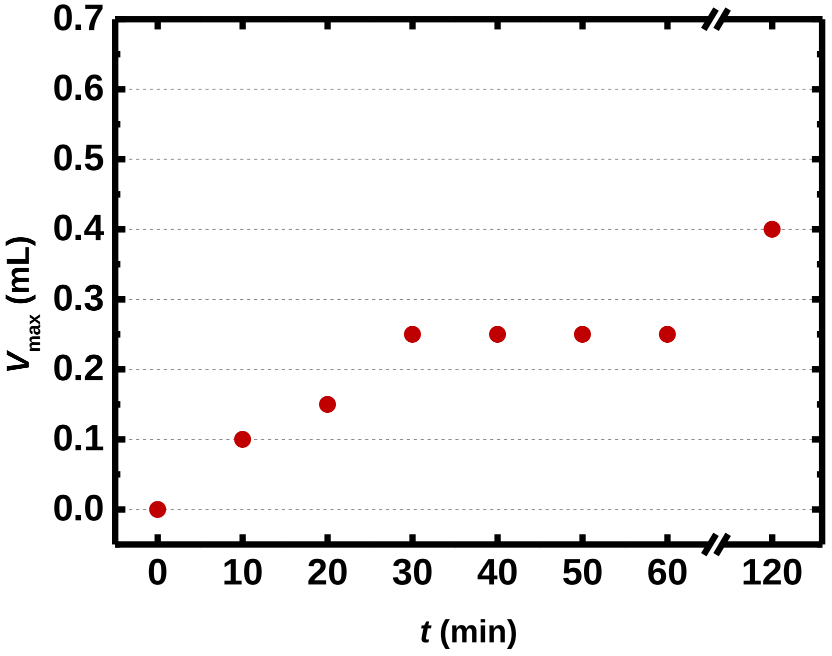

Figure S14. Swelling of a **PDCPD** aerogel thin disk in *p*-xylene *versus* time.

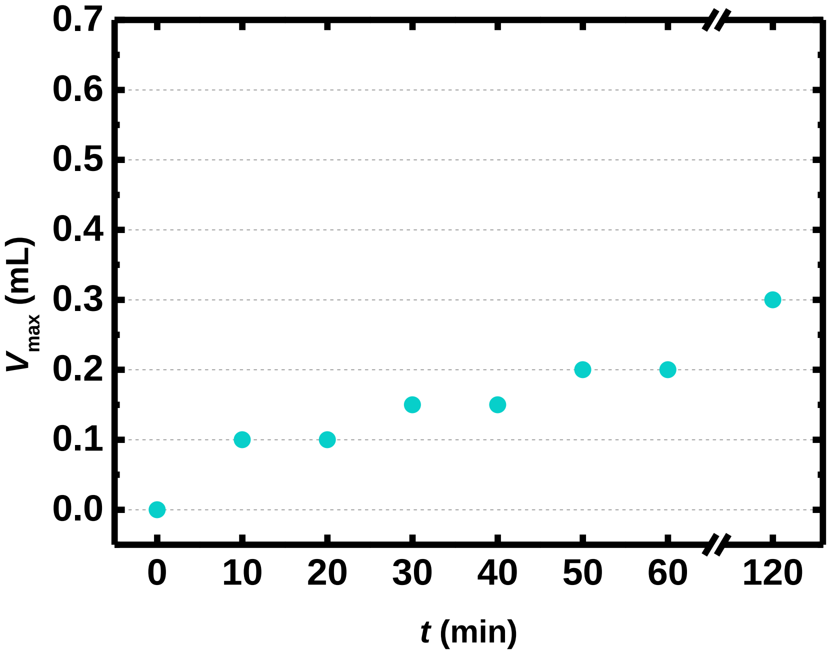

Figure S15. Swelling of a **PDCPD** aerogel thin disk in mesitylene *versus* time.
